# How can educators improve the perception of happiness for pre-clinical medical students?

**DOI:** 10.1186/s12909-020-02216-z

**Published:** 2020-09-03

**Authors:** Sami Ahmad Ghani, Muhammed Aizaz us Salam, George Chukwuemeka Oyekwe, Sharfraz Riaz Choudhury

**Affiliations:** St George’s University of London, Cranmer Terrace, Tooting, London, SW17 0RE UK

**Keywords:** Happiness, Medical student, Medical school, Problem-based learning, Work-life balance, Emotional intelligence

## Abstract

As medical students, we recognise how the various stages of medical school can influence one’s perception of their educational environment, as illustrated by the work of Yoo and Kim. Throughout this article, via the exploration of reviewed literature and personal experience, we provide a critical perspective into the significance of enhancing student happiness within pre-clinical periods of the medical curriculum. Here, we highlight the refinements and safeguards available which we believe should be taken into consideration by educators.

## Background

We thank Yoo and Kim for their research on ‘The relationship between students’ perception of the educational environment and their subjective happiness.’ This study indirectly found a positive association between medical student subjective happiness during clerkships, in contrast to their experience during their pre-medical years; all of which was respective to their medical programme [1].

As British medical students, we appreciate the contrasting educational experience that pre-clinical and clinical students may face along with the corresponding academic expectations that arise. We are of the opinion that promoting happiness cannot be understated for the mental health and wellbeing of students who undertake such an intensive degree. This belief is justified through a meta-analysis consisting of 43 countries, which demonstrated the prevalence of depressive symptoms amongst medical students to total at 27.2% - concerningly high [2].

Throughout this article, we aim to use this newly gained insight to provide a critical perspective in which student happiness and wellbeing may be fostered within the pre-clinical curriculum and maintained throughout their future careers. Here, we explore the educational environment through the direct approaches to learning as well as a wider consideration of medical school life which may affect happiness.

## Main text

Various factors are responsible for the enhancement of student happiness throughout their time at medical school, many of which are influenced by educators who construct their respective educational programmes. From our experience, we have identified the adaptation of course structure, work-life balance, and an emphasised awareness of emotional intelligence as key areas to promote happiness and therefore the long-term mental health of students. We have identified these topics in particular, as we believe each area requires greater attention from curriculum organisers to promote happiness, where adopted refinements may yield great benefits to students.

### Course structure

A students’ perception of their educational environment is largely affected by their course structure. We agree with Yoo and Kim’s assessment that pre-clinical students may be inadequately engaged through the traditional approach of didactic lectures. This can influentially reduce perceived happiness when compared to greater student engagement as a member of a clinical team within a hospital setting [1]. While a lecture-based teaching style is effective in delivering information to a large cohort of students, its predominant use as an educational tool results in a repetitive and passive style of learning. Moreover, experts believe a system that rewards rote memorisation does not prepare students for the realities of practice and deprives students of an early professional identity which may be detrimental to long-term wellbeing [3].

Across the globe, the implementation of problem-based learning (PBL) has provided students a way to learn core information that is applied to real-life scenarios. This teaching format agrees with Yoo and Kim’s view of shifting towards a ‘participation’ model which emphasises social relationships and interactions [1]. The use of real-life scenarios has been found to increase student motivation as well as stimulate a deeper learning method through encouraging individuals to interact with information on multiple levels, while in turn reducing information overload [4]. At St George’s University of London (SGUL), we apply a system that synergises the traditional lecture method with a PBL style of teaching to provide an integrated approach to learning. For instance, a cardiovascular case study during a PBL session may be complemented with the appropriate cardiovascular lectures and clinical teaching sessions throughout the week. We highly regard this pragmatic teaching method with its capacity to provide a comprehensive learning foundation to promote student satisfaction. This enables students to retain information which can be greater applied for use in future practice, rather than primarily learning for examination purposes. Moreover, this teaching style concurs with Yeo and Chang’s study regarding the impact of PBL on student satisfaction, where synchronicity with ongoing lectures can improve curricula perception [5].

### Work-life balance

We believe that medics possess a degree of perfectionism; a meticulous attention to detail to achieve a desired set of characteristics or results. It is when we do not achieve these high standards that we strive for, which may be common in a competitive environment of like-minded professionals, that feelings of inadequacy and self-criticism may lead to the detriment of one’s psychological state [6]. Yoo and Kim articulate these concerns, where the stress of pursuing academic development as a solitary goal can trigger burnout, hindering the development of professional attitudes and values [1]. We observe these pressures in a study conducted across two British universities, which observed that 54.8% of surveyed medical students reported ‘high levels of emotional exhaustion’ and 34% reported ‘high levels of depersonalisation’ which if left unresolved, can ultimately impact on future job satisfaction and patient care. Those students who engage in healthier lifestyle choices, especially physical activity, may reduce the development of burnout [7].

We understand that providing a balance to student life is essential, as literature has suggested that reduced life satisfaction can be due to educational programme interference with student social and personal life [8]. This is clarified by Yoo and Kim, who indicate that social relationships outweigh in importance for subjective happiness when compared to greater academic achievement [1]. One institution adopted a reduction of curricular hours by 10%, which assisted in lowering student-reported stress and exhaustion in addition to a greater sense of engagement, improved quality of life and mental health [2]. Likewise, at SGUL we have a free afternoon scheduled once a week to facilitate student engagement in extra-curricular activities such as sports, or to catch up with studies. It is these forms of alleviation from everyday stressors that enable students to express themselves in a non-clinical manner, providing a vital role in preventing burnout.

While a student who is solely dedicated to studying may give rise to a great clinician, we feel educators with regular student contact, such as tutors and supervisors, should take responsibility to inspire a ‘work hard, play hard’ attitude. This will give rise to a more well-rounded doctor.

### Emotional intelligence

We recognise student happiness may be attributed to a variety of environmental and personal factors. However, often overlooked is the psychology of emotional intelligence, which plays an integral role in self-perception. Characterised as a talent for understanding, feeling, and using these feelings as a basis to influence human communications, greater emotional intelligence can assist students to cope with stresses throughout their educational development [9].

While medical schools often apply measures to assist students who may experience psychological difficulties, such as counselling services or personal tutors, inadequate implementation of techniques have been achieved by medical schools to prevent student susceptibility to mental breakdown. Here, we agree that enhancing resilience can act as an innate reservoir to minimise the perception of stresses, as highlighted by Yoo and Kim [1]. We understand that providing greater accessibility to enhance this form of self-awareness through mindfulness-based wellbeing courses to improve emotional hygiene can promote resilience and in turn happiness [10]. Furthermore, formalised approaches of student-led support, such as our SGUL ‘mums and dads’ programme may be beneficial for new students, who are adopted by their senior counterparts. This programme aims to support new students throughout their time at medical school, whether it be curricular-related or within student life. Importantly, seniors can act as a ‘parental figure’ to impart valuable advice acquired from previous experience, thus providing suitable techniques and a pre-emptive awareness to tackle hardships that emerge. Such approaches are consistent with Yoo and Kim’s attentiveness towards the provision of social support to promote subjective happiness [1].

We argue it is this reinforced resilience that will act as a psychological buffer for future doctors when subjected to the inevitable stresses that a physically and emotionally demanding vocation requires. Without question, the majority of British students will be introduced into an increasingly strained National Health Service (NHS) against a background of an ageing population with complex multi-morbid conditions. This is reflected by the 77% of British Medical Association survey respondents who believed their health and wellbeing were harmed due to rising workloads [11].

Nonetheless, it should be recognised that resilience reinforcement techniques must not act as a replacement for addressing issues within the educational environment, but rather a safeguarding system to protect those who require it. Importantly, these approaches should not unintentionally comfort educators into believing they are resolving an issue, where the root of the problem remains unaddressed.

## Conclusion

Our desire to evolve the student environment through integrated course structuring, adequate work-life balancing, and greater emotional awareness, are but a few steps in which students can seek internal composure and contentment. We are in agreement with Yoo and Kim’s iteration that educators have an obligation which extends beyond an aim to produce ‘good doctors’ with adequate knowledge, skills, and attitudes [1]. Certainly, medical schools should recognise they are shaping not only the professional development of students, but also providing an opportunity to facilitate personal flourishment; both of which are necessary to support the foundations of a ‘happy doctor’. Therefore, we believe that an educator’s responsibility to cultivate academic competency through an emotionally nurturing environment must be a proactive process rather than a reactive one. For it is this holistic appreciation that we impart to our students that will ultimately be conveyed to future patients.

## Data Availability

Not applicable.

## Abbreviations

NHS: National health service
PBL: Problem-based learning
SGUL: St George’s university of London
